# Eyecare health promotion in schools around the Free State Province, South Africa: school-based interventional study

**DOI:** 10.3389/ijph.2026.1609229

**Published:** 2026-07-27

**Authors:** Xolani Nyathela, Urvashni Nirghin, Naimah Ebrahim Khan

**Affiliations:** Discipline of Optometry, University of KwaZulu-Natal, Durban, South Africa

**Keywords:** barriers, eyecare, spectacle uptake, tailored intervention, uncorrected refractive error

## Abstract

**Objectives:**

School-based health promotion is critical for enhancing health literacy and academic performance. In South Africa, preventable visual impairment among school children may be exacerbated by the absence of structured eyecare promotion interventions. The objective of the study was to implement and evaluate the effectiveness of eyecare health promotion in the Thabo Mofutsanyane district, Free State province.

**Methods:**

A school-based cross-sectional interventional study was conducted. Following a simple randomised assignment, 10 schools received the intervention while the remaining 10 received no intervention. An adopted and piloted questionnaire was administered at baseline and readministered 6 months after the intervention. The McNemar’s chi-square test was used for statistical analysis.

**Results:**

The baseline study included 199 participants, and following attrition, stood at 136 learners, parents, and teachers. The learner experimental arm demonstrated a statistically significant change in eyecare knowledge (p < 0.05). While no statistically significant changes were noted among teachers, parents and learners had statistically significant variables.

**Conclusion:**

Despite limitations, this study demonstrated that targeted eye health promotion can improve eyecare knowledge.

## Introduction

The foundation of health promotion rests on expert-driven health education, which seeks not only to influence positive eyecare behaviours but also to enhance the utilisation of healthcare (eyecare) services [1–4]. Health literacy, fostered through health promotion, plays a pivotal role in shaping health-related behaviour, which encompasses attitudes, beliefs, motivations, and values [5, 6]. For such behaviours to be sustained, particularly values, they must be coherent within broader societal norms, thereby enabling individuals to exercise greater control over their health [5, 6]. School-based health promotion has demonstrated particular value, especially in settings with a high burden of disease and limited uptake of health services, such as developing countries, including South Africa [5–7]. In these contexts, such initiatives not only enhance learners’ health literacy but also positively influence their academic performance [8]. Conversely, within the school environment, eyecare health promotion (EHP) is notably limited, with vision screening often constituting “only eye health-related activity” [9]. A lack of eyecare health promotion, especially in schools, comes as eye health is classified as imperative for quality of life [10]. This neglect arises in part because most schools' health promotion efforts have historically prioritised health aspects such as smoking cessation, physical activity, and sexual health [8, 11, 12]. School-based health promotion is most effective when addressing both the individual and the environmental determinants of health [8]. The individual component includes knowledge, behaviour, and skills, whereas the environmental dimension encompasses the social and physical environment, ranging from the family to the community [8]. A dual focus yields more sustainable behaviour change [8]. Similarly, eyecare health promotions coupled with the provision of free spectacles enhanced both knowledge of eye health and spectacle wear compliance [13–15]. The effectiveness of health promotion is further enhanced when interventions account for social and cultural concepts [8]. Participants' health, beliefs, perceived susceptibility and severity, as well as perceived benefits and barriers form part of these concepts [5].

In South Africa, the national guidelines for the prevention of blindness explicitly recognise health education as a primary prevention strategy integrated within primary healthcare [3]. Nevertheless, a review of EHP in South Africa highlighted several deficiencies: its implementation has been fragmented, largely curative rather than preventative, insufficiently prioritised, and poorly documented [2, 3]. The study determined that eyecare health promotion activities are not included in primary healthcare due to the lack of a dedicated directorate, like other disciplines [2]. These shortcomings have been attributed to weak intergovernmental collaboration and limited political will to integrate eyecare services within primary healthcare [2, 3], despite optometry being classified as a primary healthcare service [16]. This gap is particularly concerning given the centrality of vision in learning, and the profound academic, psychological, social, and economic consequences of unaddressed vision problems for children [2, 9, 17–23]. Given the significance of EHP for global health outcomes, schools, as established institutions for learning and community engagement, are well-positioned to bridge deficits in health literacy, including eye health [4, 8]. Since school communities are also susceptible to ill-health (eye problems), the provision of health services, including the promotion of health literacy, can yield good results [8]. Furthermore, teachers whose role as health educators is well recognised can play an essential role in mitigating the negative effects of vision impairment, particularly in under-resourced contexts [24, 25]. Health promotion interventions are most effective when they are culturally, socially, and behaviourally contextualised, rather than generic or misaligned with the needs of the population [5, 6].

Notwithstanding the unaffordability and inaccessibility of eyecare services for those in rural areas, the dearth of eyecare health promotion in South Africa has been attributed to poor uptake of eyecare [2]. The impetus for this study arose from observed barriers to spectacle uptake in local communities, including low levels of eye health knowledge, inaccessibility, and unavailability, factors which have been consistently reported in previous studies [5, 12, 26]. Given that several EHP interventions have demonstrated improvements in health behaviour [2, 9, 12, 22, 23, 27], It was deemed important to implement and assess the effectiveness of the intervention. This study aimed to implement an eyecare health promotion among learners, parents, and teachers and to assess its effectiveness.

## Methods

A school-based cross-sectional interventional study was undertaken between July 2023 and March 2025. Since the study design was a cross-sectional intervention, clustering was not accounted for, and the analyses were not from a cluster-randomised controlled trial. Consequently, the findings should be interpreted as exploratory and not causal evidence. The study was conducted in five stages: determine the prevalence of uncorrected refractive error among learners (July to September 2023), determine the barriers to the uptake of spectacles among learners, parents, and teachers (July to September 2023), develop (December 2023 to May 2024), validate (May to August 2024), implement (September 2024), and evaluate (March 2025) the health promotion intervention. The intervention was evaluated 6 months after its implementation, a period similar to previous school-based interventional studies [9, 15, 28, 29].

### Prevalence of uncorrected refractive error

The study commenced with a school-based cross-sectional study to determine the prevalence of URE in low-resource schools in the Free State Province, South Africa, between July and September 2023 [30]. In consultation with a statistician, a single population proportion formula (n=(Z1 − α)^2^ (P (1 − P))/d^2^) were Z1-α (the 95% Confidence Interval) of 1.96, P (Prevalence determined in a previous South African study [31]) of 0.206, and d (the margin of error, the absolute precision) of 0.05 was used to calculate the initial sample size of 1,008 learners across four districts. A stratified multistage random sampling was employed to select schools and participants. The results unveiled a prevalence of 27.1% (n = 233, 95% CI, 24.2–30.1)^30^. Thabo Mofutsanyane was the largest district and had the highest prevalence of URE (57.9%, n = 135) in the Free State province.

### Barriers to the uptake of spectacles

Upon URE diagnosis, all learners, together with their parents/guardians and class teachers, were asked to participate in a descriptive, cross-sectional survey using an adopted and piloted questionnaire. A total of 192 out of 233 learners, 132 out of 233 parents, and 23 out of 255 class teachers returned their completed questionnaires at baseline between July and September 2023. Each group of participants has their specific questionnaire adopted from previous studies [25, 32, 33]. The questionnaires covered barriers to the uptake of spectacles identified by literature as affordability, accessibility, availability, acceptability, and knowledge and/or awareness [32, 34–38]. Design and validation of the eyecare health promotion.

A clear understanding of local barriers is indispensable to effective health promotion interventions [39, 40]. The barriers phase revealed that the majority of participants failed to identify most eye problems and never had eye examinations beforehand. Participants viewed spectacles as being expensive, possibly due to the unemployment rate, while some were oblivious of where eyes are examined. Eye problems were presented at inappropriate and ill-equipped facilities, such as clinics, possibly due to their availability and easy access in every community at no cost. Eyecare services were reported to be unavailable and/or inaccessible.

### Design of the EHP

Using a methodological study, the eyecare health promotion (EHP) intervention was designed based on two guiding frameworks: the Ottawa Charter and the Health Belief Model (HBM) [12, 27]. Both these frameworks previously yielded positive outcomes [12, 27]. The HBM constructs were used to develop and validate the EHP. A total of seven questions addressed perceived susceptibility and benefits, 13 measured perceived severity, and three measured perceived barriers for both expert groups. Cues to action had six and three questions, while self-efficacy was measured by six and two questions for optometry and public health experts, respectively. The EHP materials, in collaboration with a professional graphic designer, were drafted for expert validation. The materials included a Microsoft PowerPoint presentation, pamphlets, and posters. Posters are included as Supplementary Appendix I.

A total of six experts, three from optometry and three from public health, with a minimum academic qualification of a PhD, were invited to validate the EHP using Content Validity Index (CVI). The results were consolidated on Microsoft Word, and the calculation of the I-CVI and SCVI was performed as recommended [41]. An I-CVI greater than or equal to 0.78 illustrated that the constructs were relevant to and representative of the targeted concept of promoting eyecare. The analysis achieved an I-CVI of 0.95 (Table 1). This phase was conducted between February and August 2024.

**TABLE 1:** The content validity index report (Eyecare health promotion in schools around the Free State Province, South Africa: school-based interventional study, 2025).

Item no.	Expert 1	Expert 2	Expert 3	Expert 4	Expert 5	Expert 6	Experts in agreement	I-CVI	Ua
1–35	1	1	1	1	1	1	6	1.00	1
36	1	1	1	0	1	1	5	0.83	0
37	1	1	1	1	1	1	6	1.00	1
38	1	1	1	1	1	1	6	1.00	1
39–45	1	1	1	-	-	-	3	1.00	1
Proportion relevant	1	1	1	0.75	1	1	Average proportion Relevant = 0.95		
S-CVI/Ave (average I-CVI)				​	​	​	​	0.95	-
S-CVI/UA (universal agreement)				​	​	​	​	-	0.96

I-CVI: Item content validity index.

S-CVI: Scale content validity index.

Ave: Average.

UA: Universal agreement.

Bold values represents the total number of participants for each group.

### Implementation

As aforementioned, the prevalence of URE indicated that the Thabo Mofutsanyane district had the highest prevalence, and that the majority of its schools were in quintile one (Q1), making it the most vulnerable district. Quintile one schools are classified as the poorest schools located in socioeconomically disadvantaged communities, and receive the largest proportion of government subsidies. At the same time, its learners are exempt from paying school fees [42]. As a result, this district was purposively selected for the implementation of the intervention.

All twenty schools in the district that had participated in the prevalence of URE and barriers phases were written on slips of paper and placed in a bowl. The first 10 schools randomly drawn were assigned to receive the intervention and comprised 67 learners, 52 parents, and 9 teachers. The remaining 10 received no intervention and had 42 learners, 21 parents, and 8 teachers. The questionnaires assessed participants’ knowledge, awareness of eyecare, and perceptions of eyecare in relation to accessibility, availability, affordability, and acceptability of services.

In alignment with the Ottawa Charter’s principle of service reorientation, all learner participants had undergone eye examinations. At the EHP implementation, participants were provided with spectacles at no cost. This eliminated the affordability barrier and facilitated compliance with spectacle wear. The EHP further sought to enhance personal eyecare skills and strengthen school community action through reinforcement tools, including pamphlets for participants, posters for schools and live presentations delivered by the researchers. Access to an optometrist was also established, thereby fostering a supportive environment. The intervention did not address the policy development component of the Ottawa Charter.

The treatment content included the importance and benefits of routine eye examinations: where and how to access eyecare services, the costs of obtaining these services, and visual ergonomics. The implications of blurred vision and recognition of common ocular symptoms were included. The overarching aim was to equip the participants with foundational knowledge of ocular health and promote regular eye examinations. The intervention was presented in layman’s language, supported by graphics, video clips and images. The content was delivered in either English or Sesotho, the participants’ vernacular. Similar approaches proved effective in prior studies [9, 22, 27].

**FIGURE 1:**
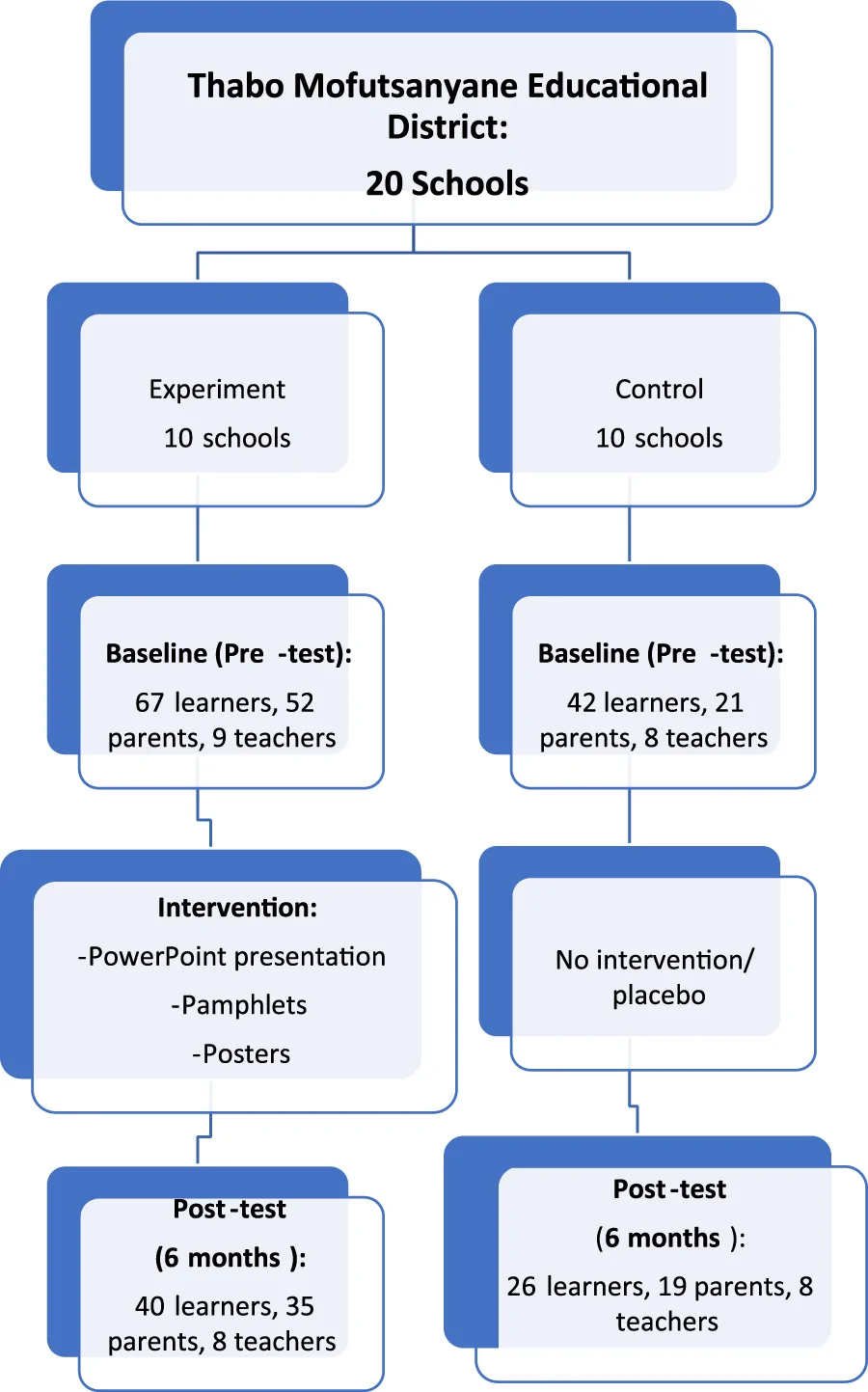
Schematic presentation of the study (Free State, South Africa. 2025).

For implementation, the researchers coordinated with the designated liaison teacher at each school. Arrangements included the school principal’s approval, securing a room large enough to accommodate all participants, and notifying parents. The presentations were projected on a large screen, followed by a question-and-answer session. Pamphlets were distributed to participants, and posters were placed in the schools. The evaluation followed established practices [9, 22, 23, 27]. Following the intervention, its effectiveness was evaluated 6 months later, in accordance with the literature [9, 15, 28, 29]. The post-intervention study was conducted on learners (n = 40), parents (n = 35), and teachers (n = 8) in the experimental group and 26, 19, and 8 learners, parents, and teachers in the control group, respectively (Figure 1). The attrition rate was 31.7% due to the unavailability of learners who had since left the respective schools.

As outlined, the intervention was designed to increase knowledge, enhance uptake of eyecare services, and ultimately reduce the prevalence of URE. Effectiveness was assessed by comparing changes in knowledge levels between the experimental and control groups from baseline to postintervention. Intervention material is provided in Supplementary Appendix I (English version only).

### Data analysis

The data were captured and analysed using the Statistical Package for Social Sciences (SPSS version 27) in consultation with a statistician. Descriptive statistics (frequency distributions, proportions, and summary tables) were used to present baseline characteristics and overall response patterns. For inferential analysis, the McNemar chi-square test was used to assess changes in paired categorical data between pre-and post-intervention responses. The level of significance was set at *p* < 0.05 for the McNemar chi-square test.

### Ethics consideration

Ethical approval for the study was obtained from the Biomedical Research and Ethics Committee of the University of KwaZulu-Natal (BREC/00005522/2023). The study was conducted in accordance with the principles of Good Clinical Practice and the Declaration of Helsinki. All participants were provided with an information sheet outlining the purpose, procedures, and requirements of the study. The document emphasised that participation was entirely voluntary, that participants could withdraw at any stage without penalty, and that all information would remain strictly confidential and anonymous. Written informed consent was obtained from all participants before enrolment.

## Results

The study had a total of 199 participants at baseline and 136 at follow-up, yielding a response rate of 68.34%. The control arm of the study had a total of 42 and 26 learners, 21 and 19 parents, and eight teachers from 10 schools in pre- and post-surveys, respectively. There were 67 learners, 52 parents, and 9 teachers in the experimental pre-survey arm, while the post-survey had 40 learners, 35 parents, and 8 teachers from the other 10 schools. At baseline, most participants were female (70.8%, n = 141), and most learners were aged 18 years (22.5%, n = 23). The proportion of learner and parent participants who either noted the importance of clear vision or became aware of their children’s eye problems increased to 100% in the post-test survey of both the control and experimental groups (Table 2).

**TABLE 2:** Awareness of eye problems and eyecare facilities (Eyecare health promotion in schools around the Free State Province, South Africa: school-based interventional study, 2025).

Question	Learner				Parent			
	Control		Experimental		Control		Experimental	
	Is clear vision important				Are you aware that your child had eye problems			
	Pre-test	Post-test	Pre-test	Post-test	Pre-test	Post-test	Pre-test	Post-test
Response	% (n)	% (n)	% (n)	% (n)	% (n)	% (n)	% (n)	% (n)
YES	95.2 (40)	100 (26)	98.5 (66)	100 (40)	95.2 (20)	100 (19)	90.4 (47)	100 (35)
NO	4.8 (2)	0	0	0	4.8 (1)	0	1.9 (1)	0
I DO NOT KNOW	0	0	1.5 (1)	0	0	0	7.7 (4)	0

Question	Where would you present your eye problems				Where would you present your child’s eye problems			
Local clinic	26.2 (11)	26.9 (7)	31.3 (21)	35 (14)	14.3 (3)	31.6 (6)	26.9 (14)	48.6 (17)
Medical Doctor	31 (13)	3.8 (1)	22.4 (15)	12.5 (5)	9.5 (2)	10.5 (2)	7.7 (4)	17.1 (6)
Optometrist	35.7 (15)	65.4 (17)	40.3 (27)	50 (20)	71.4 (15)	52.6 (10)	57.7 (30)	31.4 (11)
I don’t know	7.1 (3)	3.8 (1)	6 (4)	2.5 (1)	4.8 (1)	5.3 (1)	0	0
**Total**	**42**	**26**	**67**	**40**	**21**	**19**	**52**	**35**

Pre-test: Baseline.

Post-test: Post-Intervention.

Bold values represents the total number of participants for each group.

The learner experimental group demonstrated statistically significant (p < 0.05) within-participant pre-post-test changes for most eye-related knowledge items. The parent experimental group demonstrated statistically significant (p < 0.05) within-participant pre-post-test changes for double vision, itchy eyes, red eyes, and teary eyes. No statistically significant (p < 0.05) within-participant pre-post-test changes were noted in any of the participants in the control arm, as well as in the teacher experimental arm. The knowledge of eye-related signs and symptoms of participants in pre-and post-survey legs for both the control and experimental arms is indicated in Table 3.

**TABLE 3:** The presentation of the eyecare knowledge (Eyecare health promotion in schools around the Free State Province, South Africa: school-based interventional study, 2025).

Participant	​	​	Learner				​		Parent		​	
Arm	​	Control	​	Experimental			Control		​	​	Experimental	
​	Pre-test	Post-test	*p-*value	Pre-test	Post-test	*p-*value	Pre-test	Post-test	*p-*value	Pre-test	Post-test	*p-*value
Signs and symptoms	% (n)	% (n)	​	% (n)	% (n)	​	% (n)	% (n)	​	% (n)	% (n)	​
Blurred vision	69.2 (18)	80.8 (21)	0.453	70 (28)	87.5 (35)	0.039	78.9 (15)	78.9 (15)	1.000	74.6 (26)	88.5 (31)	0.227
Double vision	23.1 (6)	15.4 (4)	0.727	25 (10)	40 (16)	0.286	21.1 (4)	5.3 (1)	0.250	17.1 (6)	48.6 (17)	0.027
Eye strain	26.9 (7)	38.5 (10)	0.581	37.5 (15)	77.5 (31)	<0.001	42.1 (8)	36.8 (7)	1.000	74.3 (26)	82.9 (29)	0.508
Headaches	23.1 (6)	42.3 (11)	0.180	45 (18)	65 (26)	0.152	47.4 (9)	36.8 (7)	0.727	45.7 (16)	62.9 (22)	0.286
Itchy eyes	42.3 (11)	46.2 (12)	1.000	55 (22)	85 (34)	0.008	36.8 (7)	36.8 (7)	1.000	48.6 (17)	91.4 (32)	<0.001
Red eyes	30.8 (8)	38.5 (10)	0.754	15 (6)	67.5 (22)	<0.001	21.4 (4)	21.1 (4)	1.000	17.1 (6)	80 (28)	<0.001
Teary eyes	23.1 (6)	34.6 (9)	0.453	37.5 (15)	70 (28)	0.011	26.3 (5)	21.1 (4)	1.000	42.9 (15)	71.4 (25)	0.031
**Total**	**26**	**26**	​	**40**	**40**	​	**19**	**19**	​	**35**	**35**	​

Teacher						
Arm	​	Control	​	Experimental		
​	Pre-test	Post-test	*p-*value	Pre-test	Post-test	*p-*value
Signs and symptoms	% (n)	% (n)	​	% (n)	% (n)	​
Blurred vision	62.5 (5)	62.5 (5)	1.000	50 (4)	100 (8)	1.000
Double vision	25 (2)	25 (2)	1.000	50 (4)	75 (6)	1.000
Eye strain	25 (2)	37.5 (3)	1.000	37.5 (3)	100 (8)	1.000
Headaches	37.5 (3)	37.5 (3)	1.000	50 (4)	100 (8)	1.000
Itchy eyes	50 (4)	37.5 (3)	0.500	87.5 (7)	100 (8)	1.000
Red eyes	25 (2)	12.5 (1)	1.000	50 (4)	75 (6)	1.000
Teary eyes	37.5 (3)	25 (2)	1.000	50 (4)	87.5 (6)	1.000
**Total**	**8**	**8**	​	**8**	**8**	​

Behaviour of learners with eye-related signs and symptoms	Control			Experimental		
	Pre-test	Post-test	*p-*value	Pre-test	Post-test	*p-*value
	% (n)	% (n)	​	% (n)	% (n)	​
Frowns	12.5 (1)	12.5 (1)	1.000	37.5 (3)	50 (4)	1.000
Can’t write in straight lines	50 (4)	25 (2)	1.000	50 (4)	50 (4)	1.000
Struggle to see the blackboard	50 (4)	87.5 (7)	1.000	66.7 (6)	100 (8)	1.000
Short attention span	50 (4)	25 (2)	1.000	25 (2)	62.5 (5)	1.000
Isolated and shy child	25 (2)	12.5 (1)	1.000	12.5 (1)	75 (6)	1.000
Poor academic performance	62.5 (5)	75 (6)	1.000	37.5 (3)	87.5 (7)	1.000
Poor extramural performance	50 (4)	25 (2)	1.000	25 (2)	37.5 (3)	0.625
Low self-esteem and confidence	25 (2)	50 (4)	1.000	37.5 (3)	62.5 (5)	1.000
​	**8**	**8**	​	**8**	**8**	​

p-value: McNemar; Pre-test: Baseline; post-test: Post-intervention.

Bold values represents the total number of participants for each group.

Table 4 presents the participants’ responses regarding the effects of spectacles. About 89.5% parents in the post-survey control and 88.6% in the experimental group noted spectacles as a possible remedy for blurred vision.

**TABLE 4:** Participants’ perception of spectacles (Eyecare health promotion in schools around the Free State Province, South Africa: school-based interventional study, 2025).

Learner					Parent			
​	Control		Experimental		Control		Experimental	
​	Pre-test	Post-test	Pre-test	Post-test	Pre-test	Post-test	Pre-test	Post-test
Are spectacles only for old people?								
Response	% (n)	% (n)	% (n)	% (n)	% (n)	% (n)	% (n)	% (n)
Yes	2.4 (1)	0	1.5 (1)	0	0	0	3.8 (2)	2.9 (1)
No	97.6 (41)	100 (26)	94 (63)	95 (38)	100 (21)	100 (21)	92.3 (48)	97.1 (34)
I do not Know	0	0	4.5 (3)	5 (2)	0	0	3.8 (2)	0
Are spectacles bad for the eyes (can they weaken the eyes)?								
Yes	4.8 (2)	11.5 (3)	4.5 (3)	7.5 (3)	4.8 (1)	10.5 (2)	9.6 (5)	8.6 (3)
No	78.6 (33)	80.8 (21)	73.1 (49)	80 (32)	66.7 (14)	84.2 (16)	63.5 (33)	77.1 (27)
I do not Know	16.6 (7)	7.7 (2)	22.4 (15)	12.5 (5)	28.6 (6)	5.3 (1)	26.9 (14)	14.3 (5)
What are the effects of spectacle wear non-compliance?								
No effects (impact)	11.9 (5)	7.7 (2)	7.5 (5)	20 (8)	14.3 (3)	26.3 (5)	23.1 (12)	14.3 (5)
It would worsen the child’s vision	64.3 (27)	73.1 (19)	68.7 (46)	67.5 (27)	57.1 (12)	26.3 (5)	48.1 (25)	68.6 (24)
My vision will get better	9.5 (4)	0	4.5 (3)	7.5 (3)	N/A			
Poor academic performance	N/A				23.8 (5)	42.1 (8)	23 (11)	11.4 (4)
I DO NOT KNOW	14.3 (6)	19.2 (5)	19.4 (13)	5 (2)	4.8 (1)	5.3 (1)	1.9 (1)	5.7 (2)
**Total**	**42**	**26**	**67**	**40**	**21**	**19**	**52**	**35**

Teacher					Parent				​
​	Control		Experimental		Control	Experimental	Control	Experimental	​
Question	Are spectacles only for old people?				What is the remedy for eye problems?				​
Response	**% (n)**	**% (n)**	**% (n)**	**% (n)**	19 (4)	26.3 (5)	40.4 (21)	28.6 (10)	Enough sleep
Yes	0	0	11.1 (1)	0	9.5 (2)	100 (19)	5.8 (3)	17.1 (6)	Playing outside
No	100 (8)	100 (8)	88.9 (8)	100 (8)	4.8 (1)	5.3 (1)	1.9 (1)	2.9 (1)	The problems will go away on their own
I do not Know	0	0	0	0	14.3 (3)	10.5 (2)	17.3 (9)	20 (7)	Nutritious foods and supplements
Are spectacles bad for the eyes (can they weaken the eyes)?					81 (17)	89.5 (17)	80.8 (42)	88.6 (31)	Spectacles
Yes	12.5 (1)	12.5 (1)	11.1 (1)	12.5 (1)	14.3 (3)	100 (19)	17.3 (9)	14.3 (5)	Read, use TV and computer during the day
No	87.5 (7)	62.5 (5)	77.8 (7)	87.5 (7)	4.8 (1)	0	7.7 (4)	11.4 (4)	I DO NOT KNOW
I do not Know	0	25 (2)	11.1 (1)	0	**21**	**19**	**52**	**35**	TOTAL
What are the effects of spectacle wear non-compliance?					N/A				
No effects (impact)	0	0	77.8 (7)	0					
It would worsen the child’s vision	75 (6)	62.5 (5)	11.1 (1)	87.5 (7)					
Poor academic performance	25 (2)	25 (2)	11.1 (1)	12.5 (1)					
I DO NOT KNOW	0	12.5 (1)	0	0					
Total	**8**	**8**	**9**	**8**					

N/A* not applicable; Pre-test: Baseline; post-test: Post-intervention.

Bold values represents the total number of participants for each group.

## Discussion

The study aimed to implement an eyecare health promotion (EHP) and assess its effectiveness in a purposively selected district. The promotion improved the knowledge of eye-related signs and symptoms among learners and parents, thereby creating opportunities for early care-seeking behaviours and peer/family advocacy. This improvement may be attributed to the vision screening, the provision of spectacles and the EHP, substantiating the use of both the Ottawa Charter and the Health Belief Model. Several studies have indicated that EHPs accompanied by the provision of free spectacles yield enhanced eyecare knowledge among learners, parents, and teachers [13–15]. However, this study failed to assess and report the association between compliance with spectacle wear and improved eyecare knowledge. This was noted as a concern, as most EHP studies do not report this association [13]. Consequently, the paper cannot report behavioural changes due to the intervention.

Notwithstanding the EHP articulation that optometric services are unavailable at local clinics but rather at optometric practices, designated public hospitals, or at non-profit organisations, many learners and parents in the experimental group selected “local clinic” as their first point of care. This finding suggests a gap between knowledge transfer and behavioural intentions, which are likely influenced by structural barriers and healthcare seeking norms. Given that clinics represent the most proximate, cost-free, and familiar entry point to healthcare for most participants, their continued preference is understandable. This highlights a broader systemic issue, i.e., the need to integrate basic eye-care services within primary healthcare facilities to align health-seeking behaviour with service provision. The high pre-intervention acceptability of eyecare services explains the lack of statistical significance (*p* > 0.05) regarding behaviour change. Misconceptions about the potential harm of spectacles persisted in a minority, but the majority correctly rejected this belief, possibly because of cultural beliefs and health communication barriers.

Although statistical improvements regarding some dependent variables among learners and parents were noted, there was no association between dependent and independent variables. The low teacher participation rate could be attributed to the no statistically significant improvements in the identification of eye-related signs, symptoms, or child behaviours. Teachers’ involvement in EHP interventions proved indispensable in ensuring spectacle-wear compliance among learners [14]. Another study suggested that incentivising teachers should be incorporated into EHP to improve learners’ spectacle wear compliance [15]. Parental awareness of children’s visual problems also increased post-intervention, with greater recognition of symptoms such as eye rubbing and watery eyes in the experimental group than in the control group. These findings suggest that EHP may have sensitised parents to observable visual difficulties in their children, thereby increasing their vigilance.

Changes in reported remedies for eye problems were also notable. Parents in the experimental group more frequently selected outdoor activities as a preventive measure (18% post-intervention compared with 6.8% at baseline and 0% in the control group), likely reflecting exposure to the EHP’s emphasis on preventative strategies such as the “elbow rule,” “2-h rule,” and “20–2020 rule.” These behaviours are particularly relevant in the context of the ever-increasing myopia prevalence.

Overall, while the EHP did not fully resolve misconceptions or translate into significant improvements in knowledge of service accessibility, it was effective in enhancing knowledge of eye health, reinforcing positive attitudes toward spectacles, and promoting preventive strategies. This study included strategies such as video footage, pamphlets, posters, and subsidised spectacles, similar to those used in previous school-based EHP studies [7, 9, 13–15, 29]. The use of schools as a platform to implement EHP yielded positive results, as highlighted in other studies [4, 9, 15]. Additionally, EHP among school communities, which resulted in improved symptom recognition, may lead to earlier referrals and better academic outcomes.

### Strengths

A key strength of this study is its use of a tailor-made, culturally and contextually informed health promotion that was implemented at the school-community level [9, 13, 15, 29]. This enhanced the ecological validity of the findings by embedding the intervention in a real-world educational setting. The inclusion of multiple stakeholders: learners, parents, and teachers, allowed for a more holistic understanding of how eye health knowledge is disseminated and retained within communities [9, 15]. The use of well-established theories and the validation of the intervention ensured that the EHP was sound [15]. The study delivered its EHP in a manner consistent with the literature, including the 6-month follow-up period [7, 9, 13–15, 29].

### Limitations

This study is subject to several limitations. First, the relatively small sample size limits the generalisability of the findings and reduces the power to detect subtle differences across groups. Loss of participants at follow-up further exacerbated the generalisability of the findings. Second, while the EHP explicitly stated that optometric services are not available at local clinics, this message was not retained by many participants, suggesting either limitations in the clarity of communication or the overriding influence of entrenched health-seeking patterns. The reliance on self-reported data may have introduced response bias, particularly in questions regarding health-seeking behaviour.^43^ Finally, the study did not report the spectacle wear compliance, which formed part of the intervention, and thus did not report the behavioural change [13].

### Recommendations

The study was conducted among participants diagnosed with URE, their parents/guardians, and teachers. Eyecare health promotion can be broadened to include the entire school community and can be conducted annually. Future EHPs could be conducted by trained school community members, as has been proven successful in Tanzania [7]. The EHP must remain tailored to the respective communities. Eyecare services need to be brought closer to the people, in their communities. Additionally, there is a need to train teachers not only on symptoms but also on the associated psychosocial and academic impacts of vision problems. This training could be integrated into their undergraduate programme and disseminated in ongoing workshops.

### Conclusion

Notwithstanding the limitations related to small sample sizes, reliance on self-reports, and short-term follow-up, the findings support the feasibility and value of implementing eye health promotion in school settings. Although the study did not assess the subsidised spectacle wear compliance in line with the literature, it improved participants' knowledge of eye-related signs and symptoms. [15, 44] Despite these constraints, this study demonstrated that targeted eye health promotion can effectively address misconceptions, enhance symptom recognition, and promote preventative behaviours. Future interventions that are tailored and large in scale, sustained over longer periods, and supported by the integration of basic eye-care services within primary healthcare facilities are likely to achieve greater and more durable impacts.
