# Correction: Eyecare health promotion in schools around the Free State Province, South Africa: school-based interventional study

**DOI:** 10.3389/ijph.2026.1610220

**Published:** 2026-08-20

**Authors:** Xolani Nyathela, Urvashni Nirghin, Naimah Ebrahim Khan

**Affiliations:** Discipline of Optometry, University of KwaZulu-Natal, Durban, South Africa

**Keywords:** barriers, eyecare, spectacle uptake, tailored intervention, uncorrected refractive error

There was a mistake in Figure 1 as published. “Post-test” was mistakenly written as “pos- tes” in both the Intervention and Control sections of the last blocks. The intervention's last block was missing the number of months “6”. The corrected Figure 1 appears below.

**FIGURE 1:**
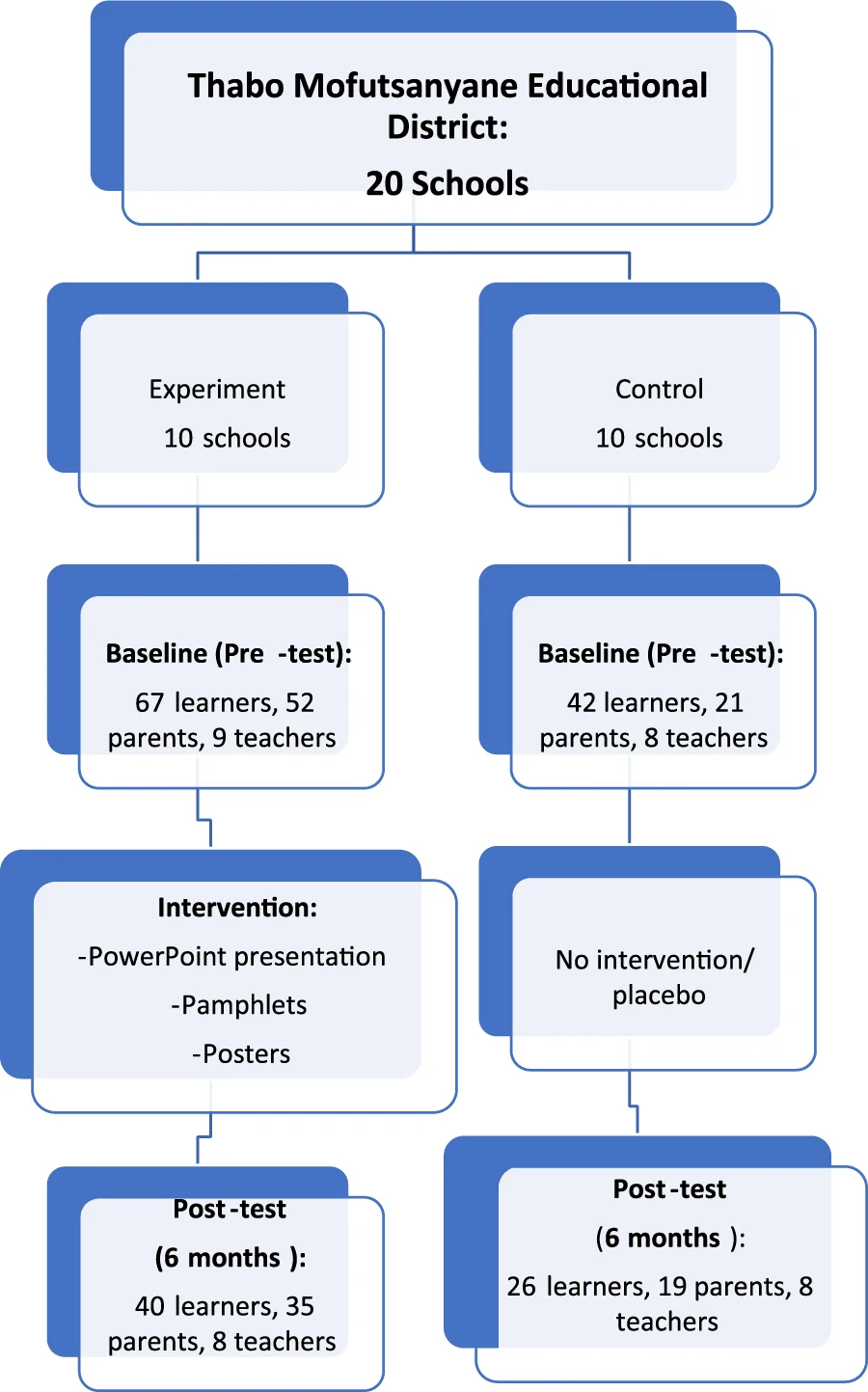
Schematic presentation of the study (Free State, South Africa. 2025).

The original version of this article has been updated.

