# Supplementary material for: Eyecare health promotion in schools around the Free State Province, South Africa: school-based interventional study
Source: Int J Public Health. 2026 Jul 27;71:1609229. doi: 10.3389/ijph.2026.1609229 (PMC13453956; doi:10.3389/ijph.2026.1609229)

Appendix I: EHP material (poster)

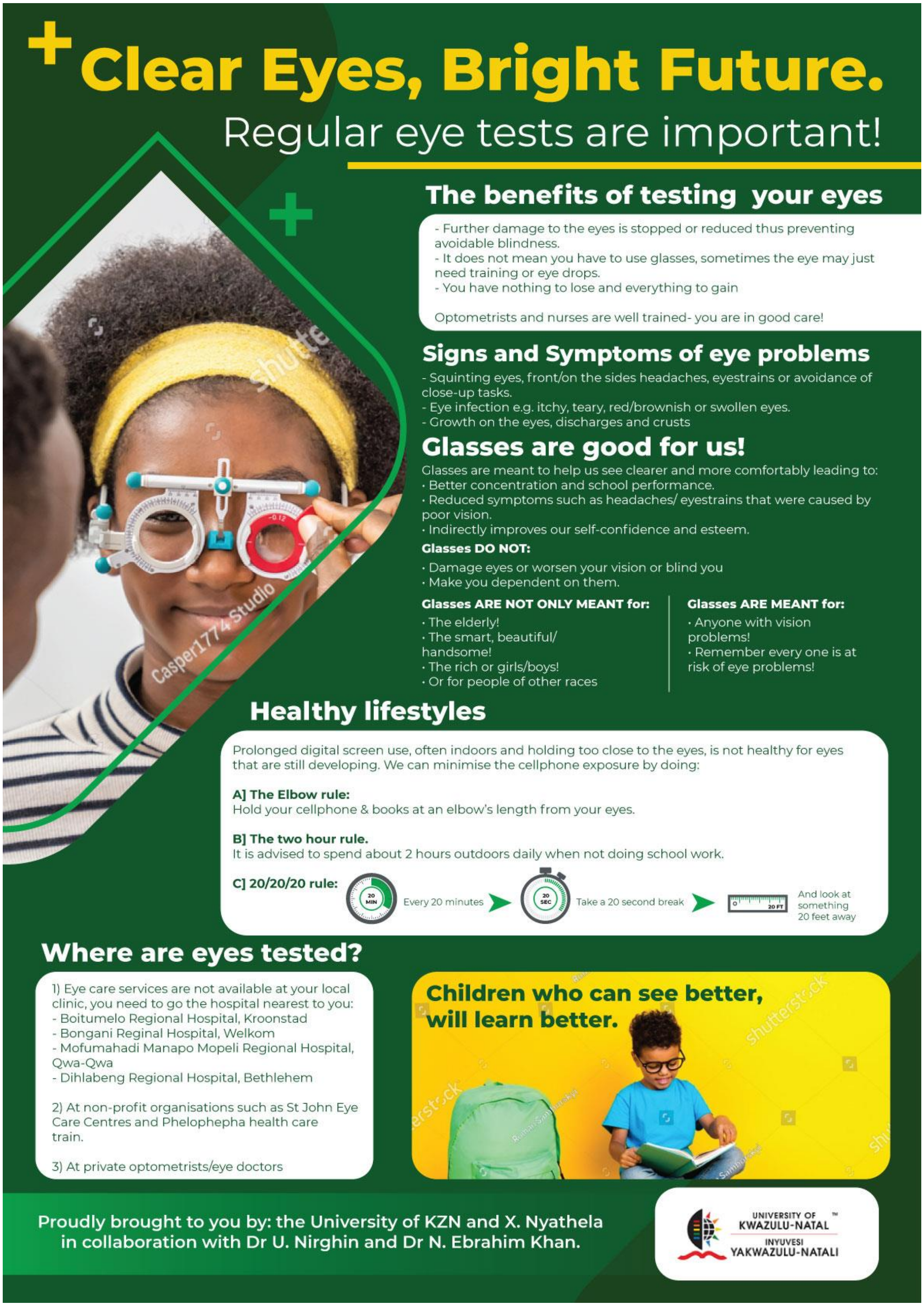

Supplement: Supplementary file 1 [file DataSheet1.pdf]
